# Antimullerian hormone is a predictor of live birth in patients with recurrent pregnancy loss

**DOI:** 10.1186/s40738-019-0054-z

**Published:** 2019-03-15

**Authors:** Gayathree Murugappan, Lora Shahine, Ruth B. Lathi

**Affiliations:** 1 Division of Reproductive Endocrinology and Infertility, Department of Obstetrics and Gynecology, Stanford Hospital and Clinics, Stanford, CA USA; 2 Pacific NW Fertility and IVF Specialists, Seattle, WA USA

**Keywords:** Recurrent pregnancy loss, Spontaneous conception, Ovarian reserve, AMH, Diminished ovarian reserve

## Abstract

**Background:**

Ovarian reserve testing is not routinely performed in the evaluation of recurrent pregnancy loss (RPL). The objective of this study was to determine if AMH levels are predictive of live birth rate in RPL patients pursuing expectant management (EM).

**Methods:**

Retrospective cohort study of RPL patients. Patients tried to conceive spontaneously for 12 calendar months or until they achieved a live birth, whichever occurred first. All patients with the intent to conceive were included regardless of final outcome.

**Results:**

One hundred fifty-five RPL patients treated from 2009 to 2017 were included. In a univariate logistic regression, AMH < 1 ng/mL was associated with decreased likelihood of live birth (OR 0.38; CI 0.16–0.87, *p* = 0.03) and increasing age (OR 0.91; CI 0.83–0.99, *p* = 0.04). Likelihood of live birth was not significantly associated with BMI (OR 1.21; CI 0.83–1.77, *p* = 0.31), three or four or more prior pregnancy losses (OR 0.93; CI 0.40–2.22, *p* = 0.87 and OR 0.52; CI 0.19–1.42, *p* = 0.20, respectively) and prior live births (OR 1.00; CI 0.48–2.08, *p* = 0.99). AMH < 1 ng/mL was shown to be a stronger predictor of live birth than age using a multivariate model adjusting for age, AMH, and time to conception.

**Conclusions:**

AMH < 1 ng/mL is associated with decreased likelihood of live birth among RPL patients pursuing EM, and may be a stronger predictor of live birth than age in this population.

**Electronic supplementary material:**

The online version of this article (10.1186/s40738-019-0054-z) contains supplementary material, which is available to authorized users.

## Background

Recurrent pregnancy loss (RPL) is a challenging disorder for both patients and clinicians [1]. The most common cause of first trimester pregnancy loss is aneuploidy within the embryo and testing the patients having miscarriages for anatomic, parental karyotypes, immune, and hormonal issues results in no abnormal findings in the majority of cases [1–5]. The American Society for Reproductive Medicine (ASRM) and the European Society of Human Reproduction and Embryology (ESHRE) recommend expectant management (EM) as the standard of care for patients with unexplained RPL [1, 2]. While ovarian reserve testing is not routinely recommended as part of the workup for RPL patients [1], this testing is recommended for women with infertility and has been shown to predict response to gonadotropin stimulation in fertility treatments like in vitro fertilization (IVF) [6, 7]. The ability of biomarkers such as AMH to predict overall reproductive potential, however, is uncertain. A large prospective cohort study of fertile women trying to conceive spontaneously found that AMH did not correlate with fecundability [8]. In a subsequent analysis of pregnancy outcomes in this cohort, the authors report that AMH levels are inversely associated with the risk of miscarriage [9]. The role of AMH in predicting reproductive potential in RPL patients, defined as having two or more prior pregnancy losses, is unknown. A prospective cohort study of women attempting spontaneous conception after one or two prior pregnancy losses found that AMH was not associated with fecundability, while live birth was not separately examined [10]. In a population of women who have experienced multiple pregnancy losses, one can argue that their ultimate goal is to achieve live birth, as opposed to clinical pregnancy. The objective of our study is to examine the role, if any, of AMH in predicting live birth rate in RPL patients attempting spontaneous conception.

## Methods

### Patients and workup

This is a retrospective cohort study of RPL patients treated at one academic fertility center and one private practice setting between 2009 and 2017. This study was approved by the Institutional Review Board of Stanford Hospital and the Western Institutional Review Board. Patients with a history of at least 2 prior pregnancy losses, defined as loss of pregnancy from conception through 20 weeks gestational age, were included. This study was designed as an intent to treat analysis, so all RPL patients attempting spontaneous conception were included regardless of final clinical outcome. All patients had a complete RPL workup as recommended by the ASRM including blood work for parental karyotypes and to detect anti-phospholipid syndrome (APS) including the presence of anti-cardiolipin antibody, lupus anticoagulant and beta-2-glycoprotein as well as a uterine cavity evaluation. All patients also underwent screening for thyroid function; and, if they reported irregular menses, had screening with prolactin levels. Unexplained RPL patients as well as patients with APS and uterine cavity anomalies were included. Patients with APS were treated with low dose aspirin and prophylactic heparin or enoxaparin. Patients with uterine cavity anomalies including a uterine septum, submucosal fibroids or endometrial polyps underwent hysteroscopy with reection of the uterine septum, myomectomy, or polypectomy, respectively. Patient with translocations (either maternal or paternal) were excluded. Patients also had ovarian reserve testing with serum AMH prior to attempting pregnancy. Diminished ovarian reserve (DOR) was defined as AMH < 1.0 ng/mL. All patients were followed for at least 12 months from the initial clinic visit.

### Treatments offered

RPL patients attempting to conceive spontaneously were offered supportive care with serum bHCG levels and first trimester ultrasounds. In addition, patients were allowed low dose aspirin treatment and vaginal progesterone supplementation in the luteal phase or with positive bHCG at the discretion of their provider. Patients tried to conceive spontaneously for 12 calendar months or until they achieved a live birth, whichever occurred first. The main study outcomes were pregnancy rate (PR), live birth rate (LBR), and clinical miscarriage rate (CMR) per pregnancy.

### Study definitions

Body mass index (BMI) was calculated as weight in kilograms divided by height in meters squared. A clinical pregnancy was defined as a serum quantitative bHCG level > 5mIU/mL and the presence of a gestational sac on transvaginal ultrasound at 6–7 weeks of gestation. Pregnancies were then followed weekly by ultrasound until transfer of care at 10 weeks gestational age. A patient with a serum bHCG level > 5mIU/mL that never progressed to a gestational sac on transvaginal ultrasound was diagnosed with a biochemical pregnancy (BC). A patient with a serum hCG level > 5mIU/mL and an extra-uterine gestational sac was diagnosed with an ectopic pregnancy. A clinical miscarriage (CM) was defined as a loss of pregnancy after a gestational sac had been identified on ultrasound and prior to 20 weeks gestational age. A pregnancy loss was defined as loss of pregnancy from conception (defined as serum quantitative bHCG level > 5mIU/mL) through 20 weeks gestational age. A live birth (LB) was defined as birth of a neonate at or beyond 24 weeks gestation and was documented by patient report. When results were not available, patients were individually contacted for follow-up. Pregnancy rate was calculated per patient while live birth and clinical miscarriage rates were calculated per pregnancy. Time to pregnancy was calculated from date of new patient visit to date of positive bHCG result in both groups, regardless of when the cause of RPL was addressed. All pregnancies included were conceived after hysteroscopy or APS treatment.

### Statistical analysis

The primary outcome for the study was live birth by 12 cycles of attempting spontaneous conception. Secondary outcomes included clinical pregnancy rate and pregnancy loss rate.

In order to compare patient demographics, the Shapiro-Wilk test was first used to determine normality of each distribution. Continuous data with a normal distribution was reported as a mean value with standard deviation. The unpaired two-tailed student t-test was used to analyze the difference between means. Continuous data with a non-normal distribution was reported as a median with inter-quartile range (IQR). The Mann-Whitney U Test was used to determine the difference between medians. Categorical data was presented as percentages and a Fisher’s exact test was used to present the differences between the two groups. A logistic regression model was then used to assess the association between AMH (assigned a categorical value for AMH < 1 ng/mL vs AMH ≥1 ng/mL) and live birth while adjusting for potential confounders and adjusting for time to pregnancy as a proxy for exposure time. To test for interaction by age, a likelihood ratio test was used to compare the fit for the model without the interaction term with that of the model with the interaction term. A Cox regression model was used to assess the association between AMH (assigned a categorical value for AMH < 1 ng/mL vs AMH ≥1 ng/mL) and clinical pregnancy and pregnancy loss, since time to conception was known for each pregnancy. Kaplan-Meier curves with 95% confidence intervals were constructed to present time to successful conception and time to any conception during the 12-month study period in patients with AMH < 1 ng/mL compared to AMH ≥1 ng/mL. Difference between the groups was assessed using a log-rank test. Analyses were performed using R version 3.5.0. All testing was 2-sided. A *p*-value of < 0.05 was considered statistically significant.

## Results

One hundred fifty-five RPL patients tried to conceive spontaneously for 12 months. 90% of patients had unexplained RPL, with 5% (*n* = 8) diagnosed with uterine anomalies and 5% (*n* = 7) diagnosed with APS. Seven patients achieved a conception twice during this 12-month interval; 4 of these patients had 2 consecutive pregnancy losses and 3 of these patients had a pregnancy loss followed by a live birth. Average age of the overall patient cohort was 35.4 ± 4.8 years, ranging from 25 to 44 years. Overall, patients had an average of 2.5 ± 1.1 prior miscarriages, 0.6 ± 0.7 prior live births and a median BMI of 23.5 kg/m2 with IQR of 6.5 kg/m2. Median AMH was 1.7 ng/mL with IQR of 0.63 ng/mL. The distributions of age and AMH are shown in Additional file 1: Figures S1, S2. 29% of patients (*n* = 47) had DOR based on AMH < 1 ng/mL. As shown in Table 1, patients with AMH < 1 ng/mL were significantly older than patients with AMH ≥1 ng/mL (*p* < 0.01, student’s T test) but did not differ in terms of BMI (*p* = 0.45, Mann-Whitney U Test), number of prior live births (*p* = 0.10, student’s T test) or number of prior pregnancy losses (*p* = 0.42, student’s T test).

**Table 1:** Baseline demographics for RPL patients pursuing expectant management, stratified by patients with AMH < 1 compared to AMH ≥1

Parameter	AMH < 1 (*n* = 47 patients)	AMH ≥1 (*n* = 108 patients)	*P*-value
Maternal Age (years) (Mean ± SD)	37.5 ± 3.8	34.7 ± 4.0	< 0.01^1^
BMI (kg/m2)^3^ (Median (IQR))	23.9 (5.2)	22.9 (5.8)	0.45^2^
Prior live births (Mean ± SD)	0.7 ± 0.8	0.5 ± 0.6	0.10^1^
Prior miscarriages (Mean ± SD)	2.6 ± 1.2	2.5 ± 1.0	0.42^1^

^1^Calculated using Student’s T-Test, 2-tailed, unpaired

^2^Calculated using Mann-Whitney U Test, 2-tailed

^3^Parameter not available for *n* = 7 patients

**Fig. 1:**
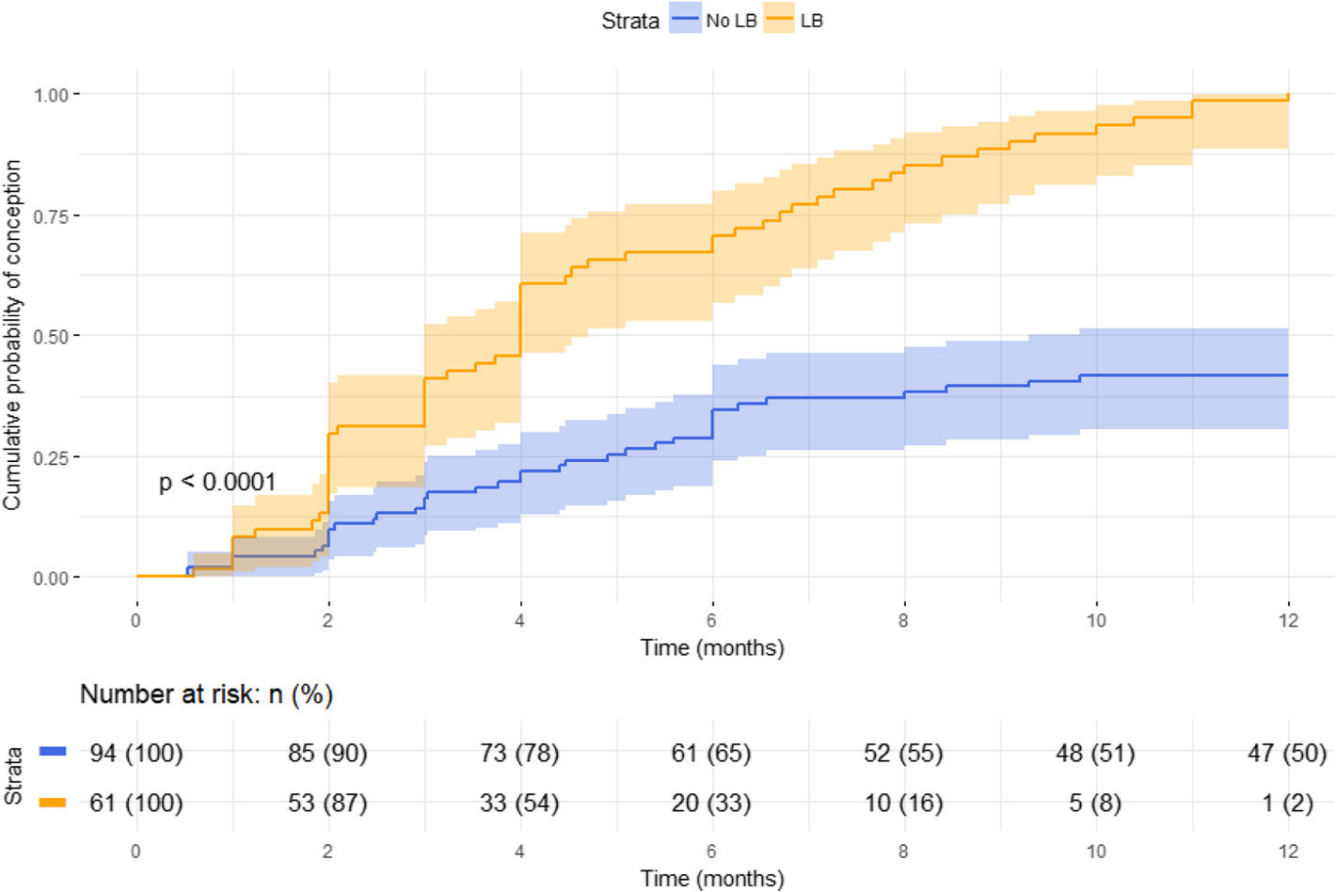
Kaplan-Meier curves for time to successful conception stratified by AMH < 1 and AMH ≥ 1 (N=155, 61 events)

Clinical pregnancy rate per patient was 66% (*n* = 103). Among patients with DOR, clinical pregnancy rate was 60% (*n* = 28). LBR per pregnancy overall was 59% (*n* = 61) and CMR per pregnancy was 40% (*n* = 41). One pregnancy was terminated for XYY karyotype on amniocentesis. Among patients with DOR, LBR per pregnancy was 39% (*n* = 11) and CMR per pregnancy was 61% (*n* = 17). Biochemical pregnancy rate per attempt was 6% (*n* = 10) and ectopic pregnancy rate per attempt was 2% (*n* = 3). Average time to pregnancy was 5.0 ± 2.8 months. Outcomes were known for all pregnancies and are stratified by AMH level in Table 2. Kaplan-Meier curves comparing time to successful conception over the 12-month study period in patients with AMH < 1 and AMH ≥ 1 are shown in Fig. 1. Patients with AMH ≥1 ng/mL had a significantly higher likelihood of achieving a successful conception (*p* < 0.01, log-rank test) compared to patients with AMH < 1 ng/mL. The shape of the curve suggests that among patients who did not succeed within the first 6 months, those with AMH < 1 ng/mL were unlikely to succeed within the following 6 months. In contrast, patients with AMH ≥1 ng/mL increased their odds of success with each subsequent month of attempting conception.

**Table 2:** Clinical outcomes for RPL patients pursuing expectant management stratified by AMH level

Outcomes by AMH level: (*n* = 155 patients)	Age (Mean ± SD)	Pregnancies per patient, N (%)	LBR per pregnancy, N (%)	CMR per pregnancy, N (%)	Time to Pregnancy, months (Mean ± SD)
AMH < 0.5 (n = 28)	37.9 ± 4.0	14 (50%)	5 (36%)	9 (64%)	4.8 ± 3.7
AMH 0.5–0.99 (*n* = 19)	36.4 ± 3.1	14 (74%)	6 (43%)	8 (57%)	4.8 ± 2.7
AMH 1–1.99 (n = 41)*	35.3 ± 6.2	30 (73%)	17 (57%)	12 (40%)	3.9 ± 2.2
AMH 2–2.99 (*n* = 24)	35.0 ± 3.9	16 (67%)	12 (75%)	4 (25%)	4.6 ± 2.3
AMH ≥ 3 (*n* = 43)	33.7 ± 3.4	29 (67%)	21 (72%)	8 (28%)	4.6 ± 3.3

*1 pregnancy was terminated for XYY karyotype on amniocentesis

A logistic regression model was then used to assess the association between AMH and live birth while adjusting for potential confounders. In order to avoid double counting, patients with more than one conception during the study period were assigned an outcome of live birth only if at least one conception during the study period resulted in live birth. In the unadjusted univariate model, AMH < 1 ng/mL was associated with decreased likelihood of live birth (OR 0.38; CI 0.16–0.87, *p* = 0.03) and increasing age (OR 0.91; CI 0.83–0.99, *p* = 0.04). Likelihood of live birth was not significantly associated with BMI (OR 1.21; CI 0.83–1.77, *p* = 0.31), three or four or more prior pregnancy losses (OR 0.93; CI 0.40–2.22, *p* = 0.87 and OR 0.52; CI 0.19–1.42, *p* = 0.20, respectively) and prior live births (OR 1.00; CI 0.48–2.08, *p* = 0.99). AMH < 1 ng/mL was shown to be a stronger predictor of live birth than age using a multivariate model adjusting for age, AMH, and time to conception (*p* = 0.14 for age and *p* = 0.07 for AMH). The interaction between AMH and age was not significant. Using a cox regression model, AMH < 1 was not found to be a significant predictor of clinical pregnancy (HR 0.72; CI 0.47–1.11, p = 0.14) or pregnancy loss (HR 1.47; CI 0.85–2.56, *p* = 0.17).

A subgroup analysis was performed comparing clinical outcomes among patients less than 38 years of age (Additional file 1: Table 1). Among patients in this subgroup, LBR per pregnancy was lower but not statistically significant between patients with AMH < 1 ng/mL compared to patients with AMH ≥1 ng/mL (47% v 69%, respectively, *p* = 0.12). Among patients in this subgroup, CMR per pregnancy was higher but not statistically significant between patients with AMH < 1 ng/mL compared to patients with AMH ≥1 ng/mL (53% v 30%, respectively, *p* = 0.09).

## Discussion

While markers of ovarian reserve such as AMH are routinely used to predict outcomes of IVF cycles in a general infertility population [6, 7], the value of AMH as an overall predictor of reproductive potential is unclear. Studies in both a fertile population and among patients with one or two prior pregnancy losses show no association between AMH and pregnancy rate, while AMH < 1 ng/mL may predict increased likelihood of miscarriage [8–10]. Among patients with RPL, one can argue that the ability to each live birth is a more significant predictor of reproductive potential than pregnancy rate. The role of AMH in predicting prognosis among RPL patients is largely unknown. In this study, RPL patients attempted spontaneous conception and clinical outcomes including live birth rate were then stratified by AMH. We report that in this RPL patient population, AMH < 1 ng/mL is associated with decreased likelihood of live birth. AMH < 1 ng/mL and may be a stronger predictor of live birth than age in this patient population, however using a multivariate model adjusting for age, AMH, and time to conception we did not find any statistically significant relationships. We did not find that an AMH < 1 ng/mL was associated with likelihood of pregnancy loss, likely because the study was underpowered to identify this association. AMH < 1 was also not associated with likelihood of clinical pregnancy, a finding that has previously been shown in an IVF population [11].

RPL patients often present a significant challenge in terms of treatment, as counseling these patients to pursue EM per clinical guidelines can feel like a passive approach. Providers may benefit from identifying a subset of RPL patients more likely to benefit from EM. Based on the results of this study, RPL patients with AMH ≥ 1 ng/mL have a favorable prognosis for achieving live birth while pursuing EM compared to patients with AMH < 1 ng/mL. Furthermore, we demonstrate that patients with AMH ≥1 ng/mL increased their odds of live birth with each subsequent month of attempting conception, while patients with AMH < 1 ng/mL who did not achieve live birth by 4 months were unlikely to succeed within the 12-month study period.

RPL patients have been noted to have lower AMH, higher serum FSH and higher percentage of DOR compared to age-matched fertile controls [12]. Furthermore, patients with unexplained RPL have a higher incidence of DOR compared to patients with an identified cause of RPL [13–15]. In this cohort of RPL patients pursuing EM, clinical miscarriage rate is higher than expected for age for RPL patients with AMH < 1 ng/mL, suggesting that ovarian reserve testing may also impact prognosis for these patients. Increased clinical miscarriage rate in patients with low AMH levels is multifactorial in etiology and may be due to decreased oocyte quality or other factors that disproportionately affect patients with diminished ovarian reserve. Since low AMH is also a negative prognostic factor for IVF outcomes in RPL patients less than 38 years of age [16], the benefit of applying preimplantation genetic testing for aneuploidy in embryos to an RPL cohort deserves further study.

To our knowledge, this is the first study examining AMH as a predictor of clinical outcomes in RPL patients pursuing EM. One attempt at EM was defined as 12 calendar months trying to conceive. We did not, however, confirm that patients were trying for each month. The structure of the study as an intent to treat analysis allows for representation of all possible clinical outcomes, both positive and negative, as they occur in clinical practice, but has limitations as noted in prior studies [14]. Livebirth outcomes were compared using logistic regression while clinical pregnancy and pregnancy loss outcomes were compared using Cox regression models because gestational age was not known for the live births, therefore time to live birth was not available for analysis. However, we used Kaplan-Meier curves as an additional descriptive summary for time to successful conception because it is a common modality to present data. Sensitivity analyses showed no difference between Cox and logistic regression models for the outcome of live birth. In an adjusted logistic regression model, we demonstrate that AMH < 1 may be a stronger predictor of live birth than age. This conclusion is limited by the age range of our patient cohort, from 25 to 44 years. In a subgroup analysis of patients less than 38 years of age, there is a trend towards AMH being predictive of LBR and CMR but the statistical analysis is limited by the small size of the cohort. While there was no significant linear interaction between age and AMH, future studies would ideally examine this effect modification in a larger cohort. Finally, the retrospective nature of data collection is a limitation as patients with less or more favorable prognoses may have chosen EM, thus introducing bias into the study results. In addition, patients were allowed treatment with aspirin and/or vaginal progesterone which is a limitation of the study inherent in its retrospective design which we could not control. Larger prospective studies are needed to verify our findings.

## Conclusion

In this intent to treat analysis, we report that AMH < 1 ng/mL is associated with decreased likelihood of live birth among RPL patients pursuing EM and may be a stronger predictor of live birth than age. Ovarian reserve testing is not currently recommended routinely in the workup of RPL. However, the findings of this study along with the higher incidence of DOR among RPL patients compared to the general infertile population may provide a more compelling argument towards screening RPL patients for DOR. RPL may be a predictor of DOR and providers will be able to counsel patients more thoroughly with ovarian reserve tests as a part of their standard RPL evaluation.

## Additional file

Additional file 1: **Figure S1.** Distribution of age (*N* = 155). **Figure S2.** Distribution of AMH (N = 155). **Table S1.** Subgroup analysis of clinical outcomes among patients less than 38 years of age. (DOCX 17 kb)

## Abbreviations

APS: Anti-phospholipid syndrome
BMI: Body mass index
CMR: Clinical miscarriage rate
EM: Expectant management
IQR: Inter-quartile range
LBR: Live birth rate
PR: Pregnancy rate
RPL: Recurrent pregnancy loss
