# Supplementary material for: Antimullerian hormone is a predictor of live birth in patients with recurrent pregnancy loss
Source: Fertil Res Pract. 2019 Mar 15;5:2. doi: 10.1186/s40738-019-0054-z (PMC6419824; doi:10.1186/s40738-019-0054-z)
Supplement: Supplementary file 1 — Figure S1. Distribution of age (N = 155). Figure S2. Distribution of AMH (N = 155). Table S1. Subgroup analysis of clinical outcomes among patients less than 38 years of age. (DOCX 17 kb) [file 40738_2019_54_MOESM1_ESM.docx]

**Supplementary Figure 1.** Distribution of age (N=155)

**Supplementary Figure 2.** Distribution of AMH (N=155)

**Supplementary Table 1.** **Subgroup analysis of clinical outcomes among patients less than 38 years of age.**

| Parameter | AMH < 1  (n=27 patients) | AMH ≥1  (n=79 patients) | P-value |
| --- | --- | --- | --- |
| Maternal Age (years)  (Mean ± SD) | 34.3±2.4 | 32.8±2.6 | 0.01^1^ |
| BMI (kg/m2)^3^  (Median (IQR)) | 24.2 (7.9) | 22.5 (5.1) | 0.21^2^ |
| Time to pregnancy (months)  (Mean ± SD) | 4.5±3.4 | 4.4±2.8 | 0.92^1^ |
| Pregnancy rate per patient | 15 (56%) | 54^4^ (68%) | 0.23^3^ |
| LBR per pregnancy | 7 (47%) | 37 (69%) | 0.12^3^ |
| CMR per pregnancy | 8 (53%) | 16 (30%) | 0.09^3^ |

^1^Calculated using ^1^Student’s T-Test, 2-tailed, unpaired

^2^Calculated using Mann-Whitney U Test, 2-tailed

^3^Calculated using Chi Squared analysis

^4^One pregnancy was terminated for XYY karyotype on amniocentesis
